# TMEM132E ablation suppresses tumor progression and restores tamoxifen sensitivity by inducing ERα expression in triple-negative breast cancer

**DOI:** 10.1016/j.gendis.2024.101396

**Published:** 2024-08-23

**Authors:** Shang Gao, Ping Sun, Zekun Wang, Yecheng Jin, Wenjie Sun, Xi Li, Ruonan Duan, Jiangxia Li, Qiji Liu

**Affiliations:** a Key Laboratory for Experimental Teratology of the Ministry of Education, Department of Medical Genetics, School of Basic Medical Sciences, Cheeloo College of Medicine, Shandong University, Jinan, Shandong 250012, China; b Department of Neurology, Qilu Hospital of Shandong University, Jinan, Shandong 250012, China; c Key Laboratory for Experimental Teratology of the Ministry of Education and Department of Medical Genetics, School of Basic Medical Sciences, Shandong University, School of Health and Life Sciences, University of Health and Rehabilitation Sciences, Qingdao, Shandong 266071, China; d NHC Key Laboratory of Birth Defects Prevention, Institute of Reproductive Health, Henan Academy of Innovations in Medical Science, Zhengzhou, Henan 451163, China

Triple-negative breast cancer (TNBC) is defined by the lack of estrogen receptor (ER), progesterone receptor, and amplified human epidermal growth factor receptor expression. TNBC accounts for ∼15% of all breast cancer cases but represents >50% of breast cancer (BC)-related mortalities.^1^ There is an urgent need for biomarkers that can predict the metastatic potential of TNBC and be used as prognostic indicators or targets for treatment. Transmembrane protein family 132E (TMEM132E, T132E) belongs to the TMEM132 family which encodes single-pass type I transmembrane proteins and consists of TMEM132A, B, C, D, and E.^2^ The *TMEM132* genes have been implicated in various cancers. Single nucleotide polymorphism association analysis suggests that *T132E* may increase the risk of BC in women undergoing menopausal hormone therapy.^3^ Few studies have explored the role of TMEM132E in BC, particularly TNBC.

BC tissue microarray datasets downloaded from The Cancer Genome Atlas (TCGA) were analyzed, the results showed that the expression of *T132E* was significantly elevated in both TNBC and non-TNBC tissues in comparison to normal breast tissues, and the same happened to TNBC tissues compared with non-TNBCs (Fig. 1A; Fig. S1A). These results were confirmed by immunohistochemistry staining applied to detect the T132E expression in BC tissue microarrays (Fig. 1B). Western blot analysis also indicated that elevated T132E expression in TNBC cells (MDA-MB-231, MDA-MB-436, and MDA-MB-468) was rather than that in normal mammary cell MCF10A and non-TNBC cells (T-47D and MCF7) (Fig. 1C). Because of the frequent amplification of T132E in patients with BC from mutation profiles and significantly shorter disease-free survival in patients with high *T132E* expression (Fig. S1B, C), T132E may play a vital role in TNBC carcinogenesis and as a potential prognostic marker for TNBC.

**Figure 1:**
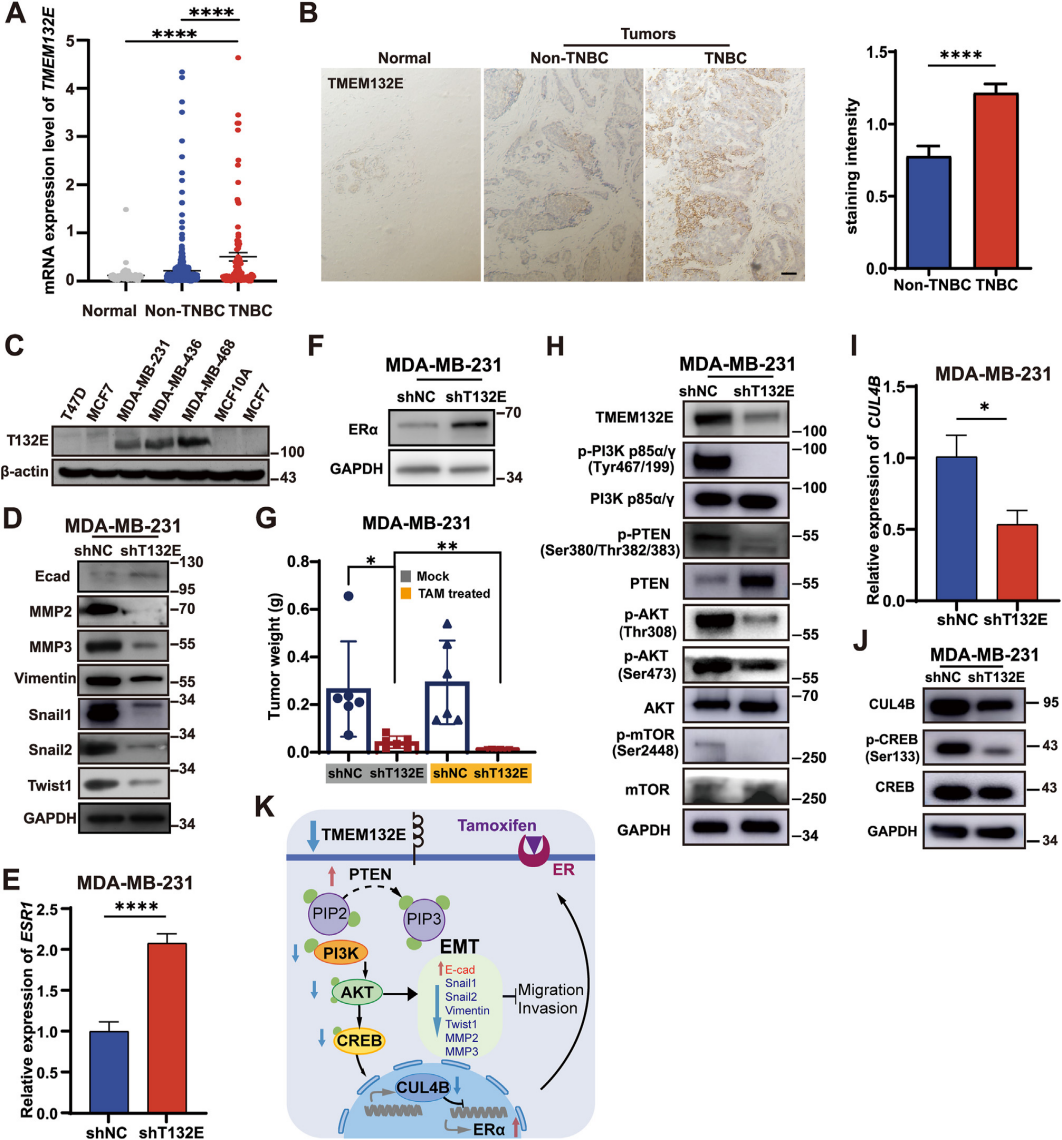
The mechanisms of TMEM132E expression in TNBC patients and cells. **(A)** The differential expression of *TMEM132E* between breast cancer patients (*n* = 554) and TNBC patients (*n* = 100) versus healthy individuals (*n* = 76) in the TCGA database. **(B)** Representative image of TMEM132E immunohistochemistry staining from tissue microarray containing non-TNBC, TNBC, and normal tissue. Staining was assessed according to its intensity, and *P* values were calculated with the Wilcoxon test. Scale bar, 50 μm. **(C)** Western blot analysis of TMEM132E expression in an assortment of breast cancer cell lines. **(D)** Protein levels of epithelial–mesenchymal transition (EMT) markers (E-cad, MMP2, MMP3, TWIST1, SNAIL1, SNAIL2, and vimentin) in MDA-MB-231 cells with shNC/shT132E treatment. GAPDH was utilized as an internal control. **(E)** RNA levels of *ESR1* in MDA-MB-231 cells with shT132E. Quantitative reverse transcription PCR was utilized to determine expression which was normalized to the vector group. The GAPDH gene was utilized as an internal control. **(F)** Immunoblot analysis for ERα and GAPDH in MDA-MB-231 cells. **(G)** Tumor weights (g) of xenograft *in vivo* in the absence and presence of T132E and TAM, respectively (*n* = 6 for each group). **(H)** Immunoblot analysis for T132E knockdown effect on multiple proteins and GAPDH expression in MDA-MB-231 cells. **(I)** RNA was extracted from control and T132E knockdown cells and quantitative reverse transcription PCR was used to analyze gene expression. Student's *t*-test. **(J)** Immunoblot analysis for CUL4B, p-CREB (Ser133), CREB, and GAPDH in control and T132E knockdown TNBC cells. **(K)** T132E deficiency both inactivated PI3K/AKT/CREB signaling and high-regulated PTEN expression, which blocks transcription of *CUL4B* RNA. Blocking *CUL4B* transcription could increase *ESR1* or ERα expression. As a result, cells became more susceptible to endocrine therapy. T132E/TMEM132E, transmembrane protein family 132E; TNBC, triple-negative breast cancer; EV, empty vector; NC, negative control; E-cad, E-cadherin; MMP2/3, matrix metalloproteinase 2/3; p-, phosphorylated; ER, indicates estrogen receptor; TAM, tamoxifen; ESR1, estrogen receptor 1; CUL4B, cullin 4B; CREB, cAMP-response element binding protein; PI3K, phosphoinositide 3 kinase; AKT, protein kinase B; PTEN, phosphatase and tensin homolog. ∗*P* < 0.05, ∗∗*P* < 0.01, ∗∗∗∗*P* < 0.0001.

To explore the role of T132E in the progression of TNBC, T132E knockdown in TNBC cell lines (MDA-MB-231 and MDA-MB-468) was achieved by short hairpin RNA (shRNA)-mediated lentiviral infection (shNC as negative control) (Fig. S1D, E). The stable overexpression of T132E in MCF7 cells was constructed by pLVX-T132E-HA lentiviral infection (T132E-HA), and an empty vector served as the control (Fig. S1F). Using the CCK-8 assay, colony formation assays, and EdU incorporation assays, we noticed that T132E knockdown significantly decreased the proliferation of TNBC cells (Fig. S2A, B, D–G, J, K), whereas the overexpression of T132E-HA in MCF7 cells promoted their proliferation (Fig. S2C, H, I, L). Flow cytometry demonstrated that T132E depletion induced more G1 arrest in TNBC cells (Fig. S2M, N), whereas T132E overexpression enhanced the G1/S transition in MCF7 cells (Fig. S2O). Therefore, these results indicated that T132E knockdown could inhibit TNBC cell proliferation by regulating the G1/S transition.

Wound healing, Transwell migration, and Matrigel invasion experiments showed that the migration and invasive capacity of T132E-silenced cells decreased markedly compared with the control cells (Fig. S3A, B, D, E). When compared with the empty vector group, the T132E-HA group increased the invasion and migratory capabilities of MCF7 cells (Fig. S3C, F). To further analyze the expression of epithelial–mesenchymal transition (EMT)-related markers to examine the involvement of T132E in the EMT, we found that T132E depletion led to a reduction in the mesenchymal markers Snail1, Snail2, TWIST1, matrix metalloproteinase 2, matrix metalloproteinase 3, and vimentin, and induction of the epithelial marker E-cadherin (encoded by CDH1/cadherin 1) (Fig. 1D; Fig. S3G). Opposing results were observed in MCF7 cells overexpressing T132E (Fig. S3G). Up-regulation of E-cadherin expression was observed in the neoplasm from the xenograft tumor model using immunohistochemistry staining, which is consistent with the negative correlation between the mRNA levels of *T132E* and *CDH1* revealed from gene expression analysis of the Breast Invasive Carcinoma dataset from TCGA and PanCancer (Fig. S3H, I). These findings suggest that T132E deficiency suppresses migration and invasion of EMT of TNBC cells.

ERα is encoded by the *ESR1* (estrogen receptor 1) gene which usually was silenced due to epigenetic mechanisms in TNBC cells. Re-expressing ERα in patients with TNBC for sensitizing tumors to endocrine therapy is a promising therapeutic strategy. The gene expression data of TCGA revealed a negative correlation between *T132E* mRNA levels and *ESR1* mRNA levels (Fig. S4A). In this study, elevated mRNA and protein levels of ERα were observed in TNBC cells following T132E depletion (Fig. 1E and F; S4B, C). When the ERα antagonist, tamoxifen, was added to the depleting T132E TNBC cells, an additive effect in inhibiting BC proliferation, invasion, and migration was observed using CCK-8 assay, wound healing, Transwell migration, Matrigel invasion experiments *in vitro*, and tumor xenografts *in vivo* (Figs. S4D–J; 1G). Thus, our findings revealed that combining T132E knockdown with ERα antagonists is a potential and effective treatment for patients with TNBC.

To explore the mechanism behind T132E in TNBC progression, transcriptome data from MDA-MB-231 cells with T132E knockdown were analyzed using RNA sequencing. Gene Ontology and Kyoto Encyclopedia of Genes and Genomes analyses revealed PI3K (phosphoinositide 3 kinase)/AKT (protein kinase B) pathway might be involved in this procession (Fig. S5A). Western blot analysis demonstrated a significant decrease in the levels of phosphorylated (p)-PI3K, p-PTEN (phosphatase and tensin homolog), p-AKT, and p-mTOR (mechanistic target of rapamycin), while an increasing PTEN level was observed in TNBC cells with T132E expression absence (Fig. 1H; S5B), which indicated that depleting T132E may inhibit the abnormally activated PTEN/PI3K/AKT/mTOR pathway in TNBC. cAMP-response element binding protein (CREB), a common downstream target of PI3K/AKT, could bind to the cullin 4B (CUL4B) gene promoter and positively regulate its transcription.^4^ CUL4B could inhibit ERα expression by binding to the ERα promoters.^5^ In our study, the RNA sequencing results from T132E-depleted MDA-MB-231 cells displayed a decrease in *CUL4B* mRNA transcription. This was confirmed by the quantitative reverse transcription PCR and western blotting results from T132E-depleted TNBC cells (Fig. 1I, J; Fig. S6A, B). Overexpression of CUL4B did not stimulate re-expression of ERα in TNBC cells with T132E silencing (Fig. S6C). Meanwhile, active p-CREB down-regulation was further observed when T132E was depleted (Fig. 1I, J; Fig. S6A, B). Therefore, TNBC cells with or without T132E expression were treated with PTEN inhibitor VO-OHpic trihydrate and PI3K activator 740Y–P, respectively. Compared with similarly treated control cells, when T132E knockdown TNBC cells were treated with VO-OHpic trihydrate and 740Y–P, the elevated p-AKT and p-CREB level, rescued CUL4B protein, and the absence of ERα demonstrated that the AKT/CREB/CUL4B pathway was activated and ERα re-expression was silenced again (Fig. S6D, E). These results demonstrate that T132E silence inhibits the proliferation, invasion, and EMT of TNBC cells by suppressing the PTEN/PI3K/AKT pathway and inducing ERα re-expression by inhibiting the AKT/CREB/CUL4B pathway in TNBC cells (Fig. 1K).

In conclusion, our results firstly indicate that TMEM132E is an oncogene in TNBC and TMEM132E depletion inhibits the proliferation, migration, invasion, and EMT in TNBC cells. Furthermore, TMEM132E depletion induces ERα re-expression. Combining TMEM132E knockdown with ERα antagonists has a synergistic therapeutic effect on TNBC cells. TMEM132E is, therefore, a promising potential diagnostic or therapeutic target for TNBC.

## Ethics declaration

All animal experiments were approved by the Institutional Animal Care and Use Committee of Shandong University (approval No. ECSBMSSDU2022-2-19). All animal housing and experiments were conducted in strict accordance with the institutional guidelines for the care and use of laboratory animals.

## Conflict of interests

The authors declare that they have no comepeting interests.

## Funding

This work was supported by grants from the National Key R&D Program of China (No. 2022YFC2703701 to Qiji Liu), the National Natural Science Foundation of China (No. 82271901, 32070586), the Shandong Provincial Natural Science Foundation, China (No. ZR2020MH086), Taishan Scholar Program of Shandong Province (tsqn202211318), and NHC Key Laboratory of Birth Defects Prevention, China (No. ZD202101).

## References

[1] Morris G.J., Naidu S., Topham A.K. (2007). Differences in breast carcinoma characteristics in newly diagnosed African-American and Caucasian patients: a single-institution compilation compared with the National Cancer Institute's Surveillance, Epidemiology, and End Results database. Cancer.

[2] Sanchez-Pulido L., Ponting C.P. (2018). TMEM132:an ancient architecture of cohesin and immunoglobulin domains define a new family of neural adhesion molecules. Bioinformatics.

[3] Rudolph A., Hein R., Lindström S. (2013). Genetic modifiers of menopausal hormone replacement therapy and breast cancer risk: a genome-wide interaction study. Endocr Relat Cancer.

[4] Ashok C., Selvam M., Ponne S., Parcha P.K., Raja K.M.P., Baluchamy S. (2020). CREB acts as a common transcription factor for major epigenetic repressors; DNMT3B, EZH2, CUL4B and E2F_6_. Med Oncol.

[5] Huang W., Zhang J., Huo M. (2021). CUL4B promotes breast carcinogenesis by coordinating with transcriptional repressor complexes in response to hypoxia signaling pathway. Adv Sci.

