# Supplementary material for: TMEM132E ablation suppresses tumor progression and restores tamoxifen sensitivity by inducing ERα expression in triple-negative breast cancer
Source: Genes Dis. 2024 Aug 23;12(2):101396. doi: 10.1016/j.gendis.2024.101396 (PMC11585717; doi:10.1016/j.gendis.2024.101396)
Supplement: Multimedia component 1 [file mmc1.docx]

**Appendix**

**Supplementary Methods**

**Cell cultures and reagents**

BC cells were purchased from the American Type Culture Collection (ATCC, USA). HEK-293T, MCF7 cells, MDA-MB-231, and MDA-MB-468 cells were maintained according to the ATCC’s instructions. The HEK-293T cell line was used for generating lentiviral particles. To prepare for transduction, polybrene (8 μg/ml; Beyotime, China) was added to the viral particle-containing medium. The PI3K activator (740 Y-P) and inhibitor (VO-OHpic) were purchased from MedChemExpress (MCE). To determine the function of Cullin 4B (CUL4B), we transfected TNBC cells with pcDNA3.1A and CUL4B pcDNA3.1A-CUL4B plasmids utilizing the TurboFect Transfection Reagent (Thermo Fisher Scientific, USA) according to the manufacturer’s instructions. The short hairpin RNA (shRNA) sequences were purchased from Shanghai GeneChem (China) and are listed in **Table S1**. The cells were treated with 2 µM Tamoxifen (TAM; Sigma-Aldrich; Merck KGaA, Darmstadt, Germany).

**RT-qPCR and western blot**

RT-qPCR and blot analysis were performed as described in our previous study ^1^. The antibodies and primers used in this study are listed in **Table S2** and **Table S3**, respectively.

**Proliferation assays**

Cells (1×10^3^) were cultured in 6-well plates for ~14 days and then stained with crystal violet to examine colony formation. Cell Counting Kit-8 (CCK-8; MCE) was used to measure cell proliferation. Briefly, the cells and culture medium (100 μL) were inoculated onto the 96-well plates (2×10^3^ cells per well) and incubated for 6, 24, 48, 72, and 96 h. After incubation, the medium was withdrawn, 10 μL of CCK-8 solution was added to the culture medium, and the cells were incubated at 37°C for 2 h. At 450 nm, optical density (OD) was measured with a microplate spectrophotometer (Thermo Fisher Scientific). Cells treated as specified were inoculated in 24-well plates and grown on glass slides. A 5-ethynyl-20-deoxyuridine (EdU) assay (RiboBio, China) was performed (according to the manufacturer’s instructions).

**Wound healing assay**

5×10^5^ transfected cells were seeded into 6-well plates. At 100% cell confluence, a 200 µL sterile pipette tip was used to draw a straight line across the center of the well, and the cells were then rinsed with phosphate-buffered saline twice. A serum-free medium supplemented with 0.1% bovine serum albumin was introduced. The width at 0 h was recorded photographically. After 48 h, wound healing was fixed, rinsed, stained with crystal violet, and observed through a microscope.

**Migration and invasion assay**

The measurement of cellular invasiveness (2.5×10^4^ cells per well) across reconstituted basement membranes was conducted using Transwell inserts (BD Biosciences, USA) with a pore size of 8 μm and coated with Matrigel (BD Biosciences). The cells were utilized at a concentration of 5×10^4^ cells per well to detect cell migration. The initial cell number was twice the number of cells when the 72h observation was used. After 48 and 72 h, migrating cells on the lower membrane surface in the upper chamber were stained with crystal violet.

**Flow cytometric analysis of the cell cycle and apoptosis**

Each experimental group's cells were obtained, fixed with ice-cold 70% ethanol at 4°C overnight, and then detached. At room temperature, cells were centrifuged at 150×g for 5 min before being suspended in 500 μL of propidium iodide (100 ng/μL) for 30 min. For the aim of detecting apoptosis, cells from each group of experiments were gathered, prepared following the instructions in the Annexin V-Fluorescein Isothiocyanate Apoptosis Detection kit I manual from BD Biosciences, and then analyzed by FACScan flow cytometry using FlowJo.7.6.2 software from the same manufacturer.

**Mouse xenograft models**

Female BALB/c nude mice (4 weeks old) were purchased from the Vital River Laboratory Animal Technology Co. Ltd, Beijing, China. All animal experiments were performed per the guidelines of the Institutional Animal Care and Use Committee of Shandong University using the authorized protocols. The cells (1×10^7^/mice, n = 6) were injected subcutaneously into the fourth mammary fat pad of 5-week-old mice. When the mean tumor volume reached approximately 100 mm^3^, the mice in control (shNC) and TMEM132E knockdown (shT132E) groups were injected intraperitoneally with or without TAM (15 mg/kg) every 3 or 4 days from day 15 to day 24. The body weight of mice and tumor volumes were measured every 3 days. The tumor volume was calculated as 0.5 × length × width^2^. The mice were euthanized 24 days later, and the tumors were collected and photographed. Tissues were analyzed by immunohistochemistry (IHC) staining using E-cadherin or MMP2 primary antibodies (as per the manufacturer’s instructions).

**Tissue microarrays**

Formalin-fixed paraffin-embedded BCs (120) and para-carcinomas (30) were used to create the tissue microarrays (Cat#HBre-Duc060CS-02 & HBreD090Bc01, Shanghai Outdo Biotech Company, China).

**mRNA sequencing**

Illumina NovaSeq 6000 sequencer paired-end readings were quality-controlled by Q30. After 3' adaptor-trimming and low-quality read removal using cutadapt (v1.9.3), hisat2 (v2.0.4) matched high-quality clean reads to the reference genome (UCSC HG19). Based on the Ensembl gtf gene annotation file, cuffdiff software (part of cufflinks) was used to generate gene-level FPKM as mRNA expression profiles, fold change, and p-value to identify differentially expressed mRNAs. The data generated in this study are publicly available in Sequence Read Archive (SRA) at PRJNA1026325.

**Pathway enrichment**

Database for Annotation, Visualization, and Integrated Discovery (DAVID)^2^ was used to conduct the pathway enrichment analysis. The Gene Ontology terms (GOTERM_BP_DIRECT)^3,4^ and Kyoto Encyclopedia of Genes and Genomes (KEGG) ^5^ were selected for biological process analysis. Separate enrichment analyses were conducted on the differentially expressed genes (DEGs) with a fold change ≥ 2. To ascertain the BC-specific mRNA-regulated pathways, the mRNA target genes were enriched by using all significant DEGs (false discovery rate < 0.05) as background.

**Statistical analysis**

All experiments were conducted with no fewer than three repetitions. The findings are presented in terms of the mean standard deviation (SD) or standard error of the mean (SEM). Student's *t*-test was used to compare quantitative data between the two groups (where *P* < 0.05 is regarded as significant). The disease-free survival (DFS) rates of the two groups were compared using Kaplan-Meier survival analysis and log-rank significance tests. The statistical analyses were conducted using GraphPad Prism (version 9.3; GraphPad Software Inc., USA).

**Reference**

1. Duan RN, Yang CL, Du T, et al. Smek1 deficiency exacerbates experimental autoimmune encephalomyelitis by activating proinflammatory microglia and suppressing the IDO1-AhR pathway. *J Neuroinflammation*. 2021;18(1):145. doi:10.1186/s12974-021-02193-0

2. Sherman BT, Hao M, Qiu J, et al. DAVID: a web server for functional enrichment analysis and functional annotation of gene lists (2021 update). *Nucleic Acids Res*. 2022;50(W1):W216-W221. doi:10.1093/nar/gkac194

3. Aleksander SA, Balhoff J, Carbon S, et al. The Gene Ontology knowledgebase in 2023. *Genetics*. 2023;224(1). doi:10.1093/genetics/iyad031

4. Ashburner M, Ball CA, Blake JA, et al. Gene Ontology: a tool for the unification of biology. *Nat Genet*. 2000;25(1):25-29. doi:10.1038/75556

5. Kanehisa M, Goto S. KEGG: kyoto encyclopedia of genes and genomes. *Nucleic Acids Res*. 2000;28(1):27-30. doi:10.1093/nar/28.1.27

**Supplemental Figures**

**
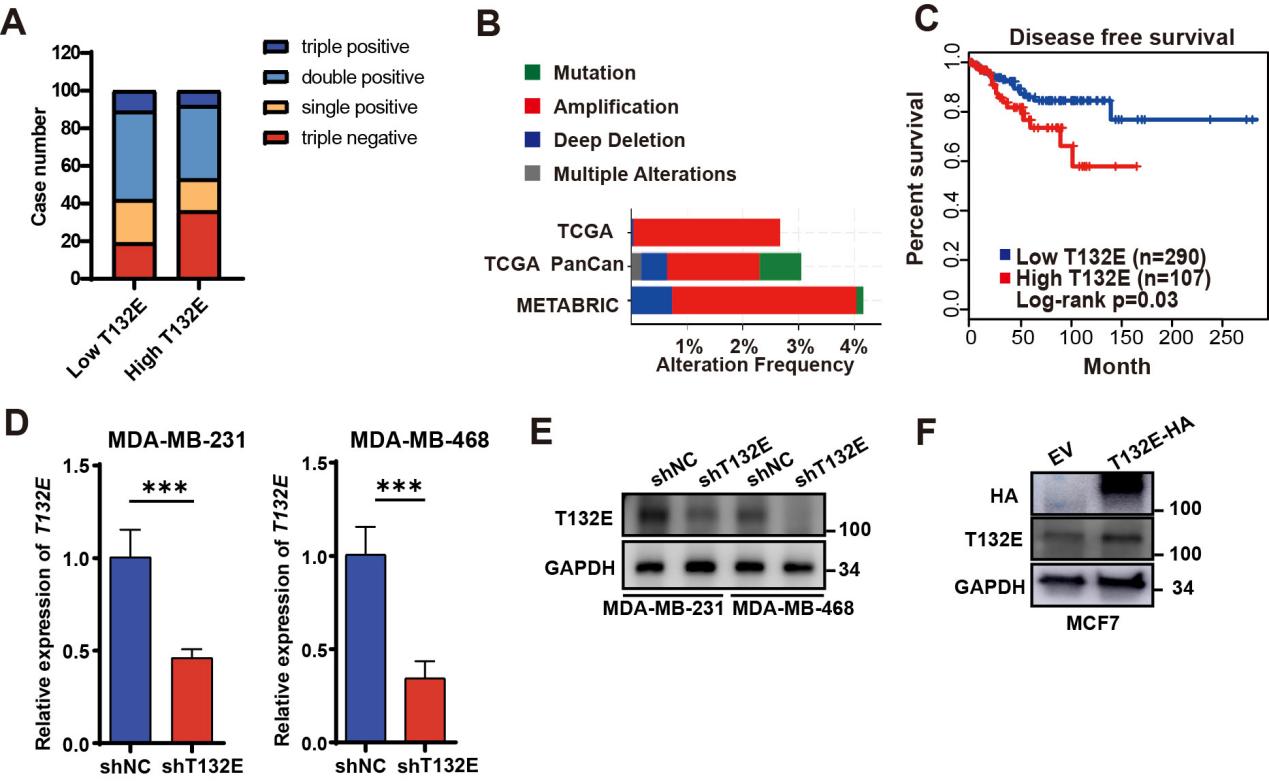
**

**Fig.S1 The expression of TMEM132E in TNBC.**

**A** TCGA database comparison of the expression of *T132E* in 200 cases with distinct breast tumor receptor subtypes.

**B** Mutation information of *T132E* in BRCA from the online databases.

**C** Kaplan-Meier curve and log-rank test were conducted to assess the effects of T132E expression on the disease-free survival of patients with breast cancer in the TCGA database (n =397). Significance was determined by the Log-rank test.

**D E** The knockdown efficacy of T132E was verified through the utilization of RT-qPCR and Western blot analysis. Data are mean ± SEM; ***p < 0.001, Student’s *t*-test (two-sided).

**F** The overexpression efficacy of T132E was verified through the utilization of Western blot analysis.

“T132E” indicates TMEM132E, “EV” indicates empty vector, and “NC” indicates negative control.

**
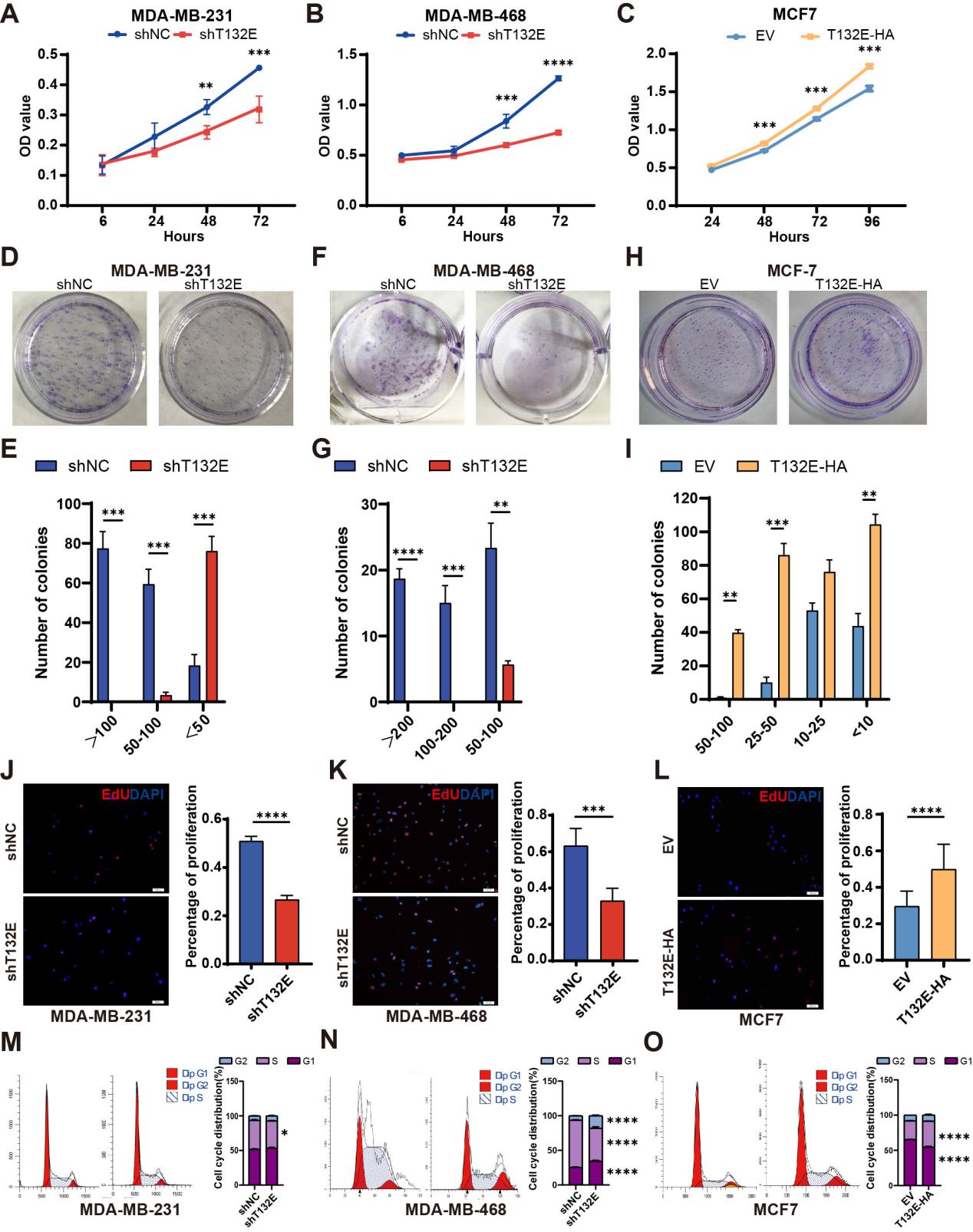
**

**Fig.S2 TMEM132E promotes the proliferation of TNBC cells *in vitro*.**

**A B** Effects of T132E suppression on cell proliferation by CCK-8 assays in MDA-MB-231 and MDA-MB-468 cells from 0 to 72 h. Absorbance is expressed as the mean ± SEM of three independent experiments.

**C** Effects of T132E overexpression on cell proliferation by CCK-8 assays from 0 to 96 h in MCF7 cells. Absorbance is expressed as the mean ± SEM for three independent tests.

**D E F G** TNBC cell lines were subjected to a colony formation assay to evaluate cell proliferation.

**H I** MCF7 cells were subjected to a colony formation assay to evaluate cell proliferation.

**J K** T132E knockdown suppressed TNBC cell proliferation, as confirmed by the EdU staining assay. The number of proliferating cells was quantified using ImageJ software. Positively stained cells were identified by red color. Scale bar: 50μm.

**L** T132E overexpression enhanced MCF7 cell proliferation, as confirmed by the EdU assay. ImageJ was utilized to count the number of proliferative cells, with positively stained cells being indicated by the color red. Scale bar: 50μm.

**M N** T132E knockdown induced cell cycle arrest in the G1/S phase, as conducted by FACS analysis in MDA-MB-231 and MDA-MB-468 cells with and without T132E knockdown.

**O** T132E overexpression promoted the G1/S transition, as conducted by FACS analysis in control or T132E overexpression MCF7 cells.

*P < 0.05; **P < 0.01; ***P <0.001; **** P < 0.0001 by Student's *t*-test.

**
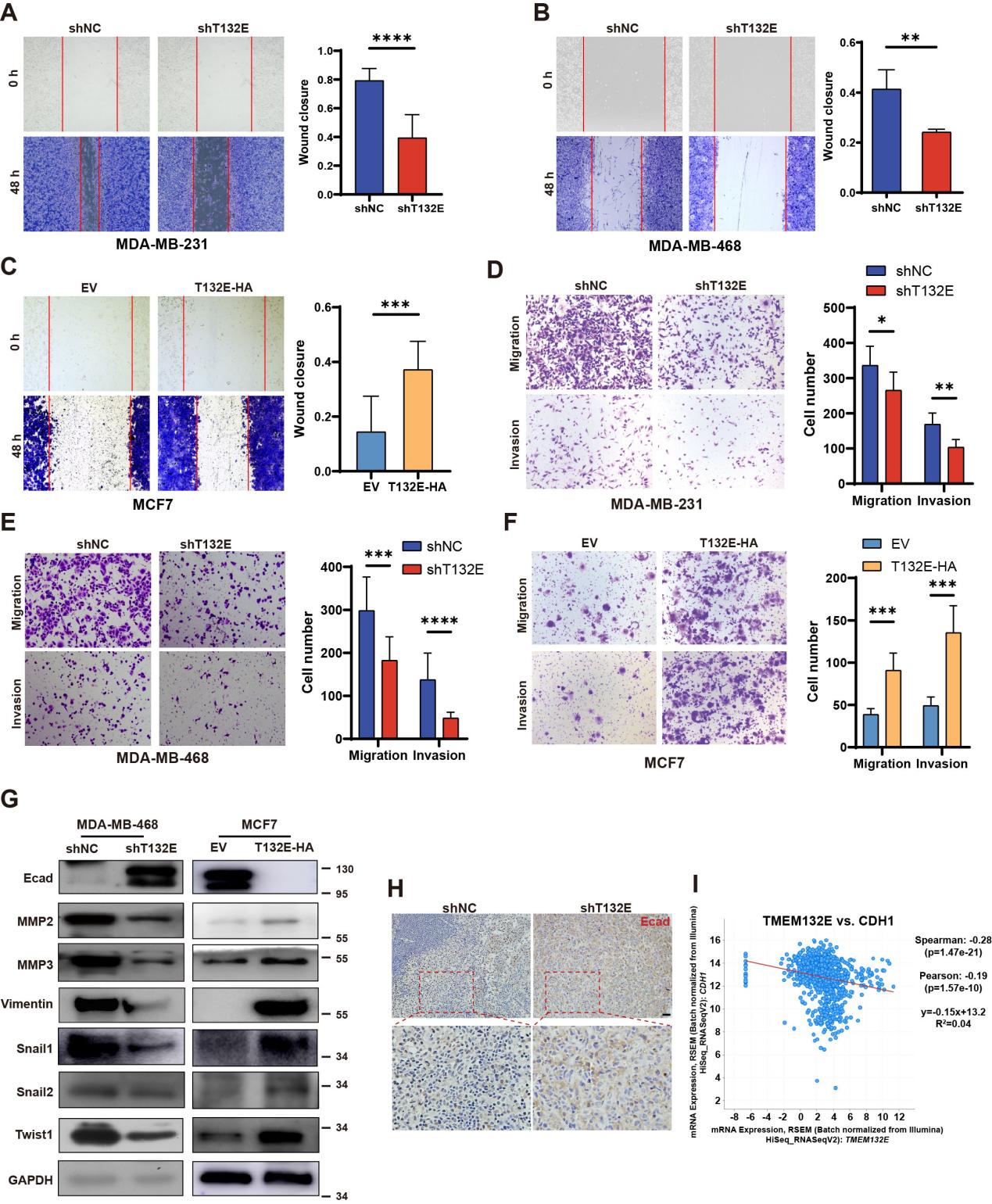
**

**Fig.S3 TMEM132E enhances migration and invasion of TNBC cells.**

**A B** Representative images of the wound healing assay to measure the migration capacity of control and T132E knockdown MDA-MB-231 and MDA-MB-468 cells at two-time points (0, 48 h) under a magnification of 4×.

**C** Representative images of the wound healing assay used to assess the migratory capability of control and T132E-overexpressing MCF7 cells at two time points (0, 48 h), magnification 4×.

**D E** Representative images of Transwell assays used to analyze and quantify the migration and invasion abilities of T132E shNC or T132E knockdown breast cancer cells (MDA-MB-231 and MDA-MB-468; 10× magnification). The quantitative bar graphs of Transwell chamber migration and invasion assay of control and T132E knockdown TNBC cells at 48 h.

**F** Representative images of Transwell assays used to analyze and quantify the migration and invasion abilities of control and T132E overexpression MCF7 cells at 48 h.

**G** Protein levels of epithelial-mesenchymal transition (EMT) markers (E-cad, MMP2, MMP3, TWIST1, SNAIL1, SNAIL2, and Vimentin) in MDA-MB-468 cells with shNC/shT132E treatment. Changes in the EMT-related proteins were detected by Western blot in MCF7 cells. GAPDH was utilized as an internal control.

**H** Representative illustrations of IHC staining for E-cadherin (E-cad) in xenograft tumor specimens with or without T132E expression. Magnification: 20×.

**I** Correlation plot showing *T132E* and *CDH1* in breast cancer TNBC subtype. Pearson pairwise correlation coefficient reveals a negative correlation (r=-0.28) between *T132E* and *CDH1* that is statistically significant (P<0.0001).

Columns represent the average quadruplicate readings of samples. Data are mean ± SEM;*P < 0.05; **P < 0.01; ***P <0.001; **** P < 0.0001, Student's *t*-test.

“Ecad” indicates E-cadherin.

**
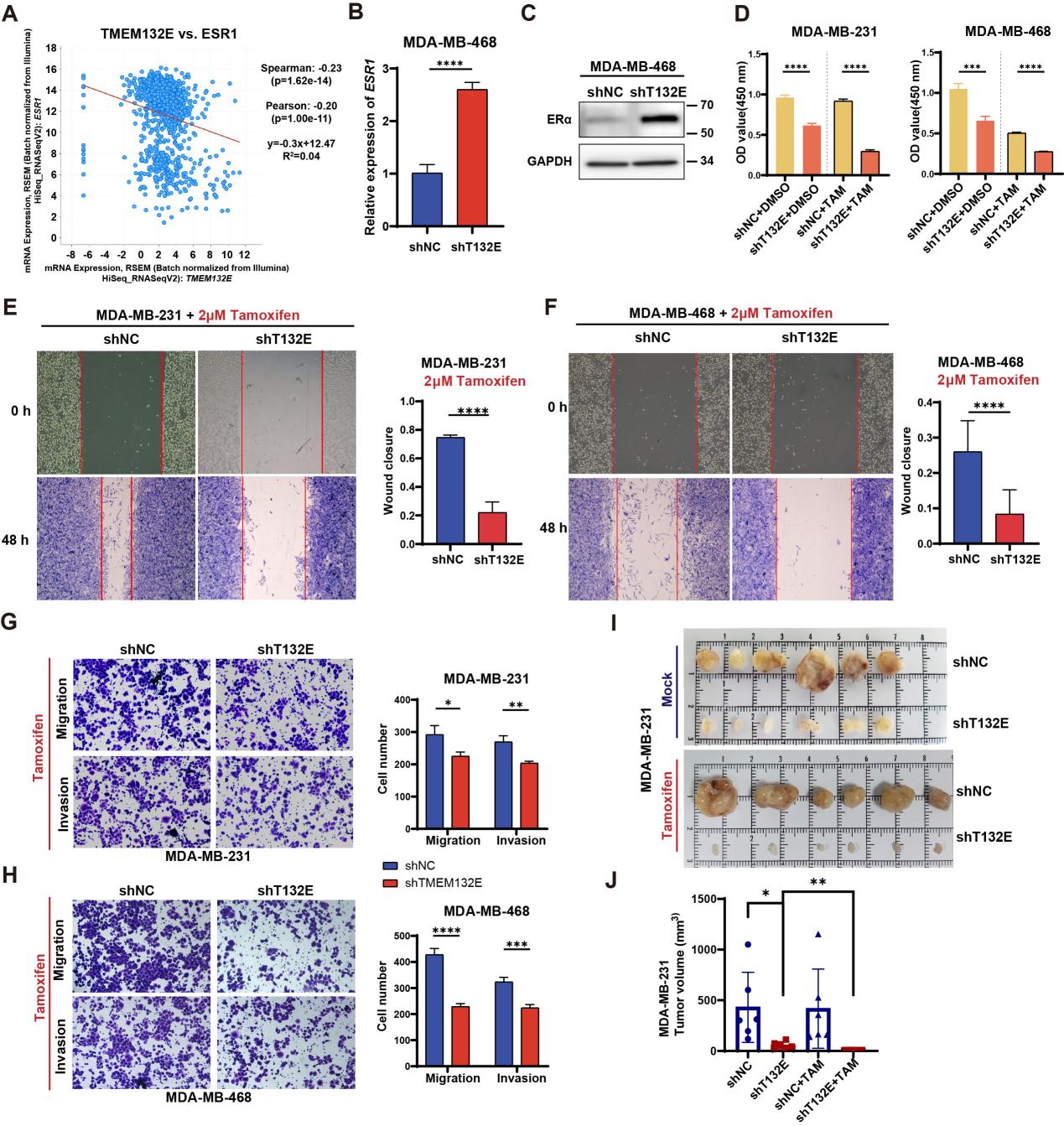
**

**Fig.S4 TMEM132E depletion compensates ERα deficiency against TNBC formation.**

**A** Correlation plot showing *T132E* and *ESR1* in TNBC. Pearson pairwise correlation coefficient between *T132E* and *ESR1* demonstrates a negative correlation (r=-0.23) and a significant association (P<0.0001).

**B** RNA levels of *ESR1* in TNBC with shT132E or T132E overexpression treatment, respectively. RT-qPCR was utilized to determine expression, normalizing to the vector group. The GAPDH gene was utilized as an internal control.

**C** Immunoblot analysis for ERα, GAPDH in MDA-MB-468 cells.

**D** Proliferative of shNC and shT132E TNBC cells with 2μM Tamoxifen treatment. Proliferation was determined by CCK-8 assay. Levels were normalized to shNC, day 0. Absorbance is expressed as the mean ± SEM of three independent experiments.

**E F** Representative images of the wound healing assay to measure the migratory capacity of TNBC cells with and without T132E knockdown at two-time points (0, 48 h), magnification 10×.

**G H** Representative images of transwell chamber assay to evaluate the migration and invasion ability of TAM (2 μM) treated shNC and shT132E TNBC cells at 72h. The quantitative bar graphs of control and TAM (2 μM) transwell chamber migratory and invasive capacities at 72 h.

**I** Representative images of xenograft tumor formation *in vivo*. Subcutaneous injection of shNC, shT132E cells was performed in the fourth mammary fat pad of BALB/c nude mice (n = 6 for each group), and the resulting tumors were grown to an average of 100 mm^3^ after inoculation. The animals were then randomized to intraperitoneally injected with or without TAM (15 mg/kg, n = 6 for each group). Mice were euthanized after 24 days.

**J** Tumor volumes (mm^3^) were measured after 24 days (n = 6 mice in each group). Student's *t*-test. Columns represent the average of duplicate measurements; error bar ± SD.

P values are determined in comparison to the control group. *P < 0.05; **P < 0.01; ***P <0.001; **** P < 0.0001.

“TAM” indicates Tamoxifen.


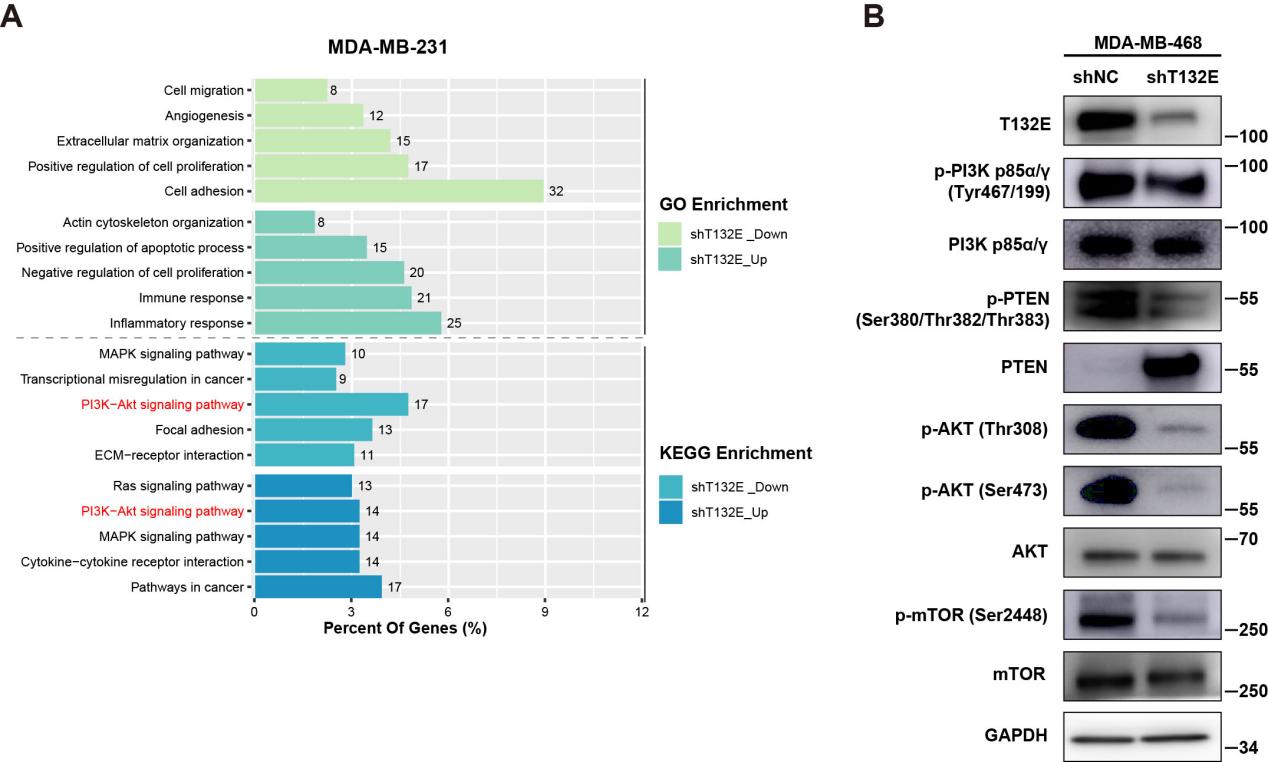


**Fig.S5 TMEM132E deficiency weakens oncogenic proliferative signaling in TNBC cells.**

**A** Significantly enriched the Gene ontology (GO) annotations and Kyoto Encyclopedia of Genes and Genomes (KEGG) and the pathways of *T132E* in the BRCA cohort. The magnitude of the bars corresponds to the gene number, while the color signifies the expression of *T132E*.

**B** Immunoblot analysis for T132E knockdown effect on multiple proteins and GAPDH expression in MDA-MB-468 cells.

“p” indicates phosphorylated.


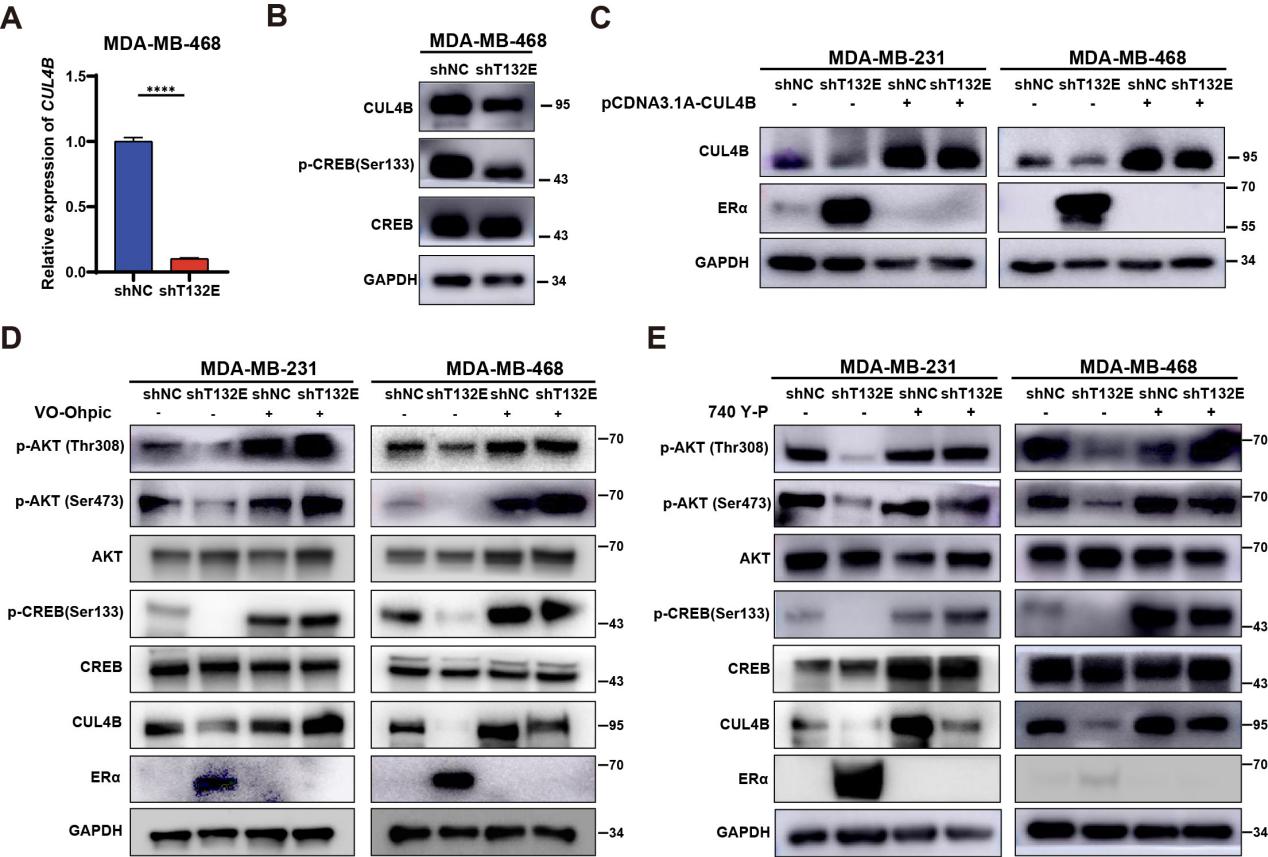


**Fig.S6 TMEM132E activated PI3K/AKT/CREB signaling and downstream CUL4B** **to promote breast cancer progression.**

**A** RNA was extracted from control and T132E knockdown cells and RT-qPCR was used to analyze gene expression. Data are mean ± SEM; ****p < 0.0001, Student’s *t*-test (two-sided).

**B** Immunoblot analysis for CUL4B, p-CREB(Ser133), CREB, GAPDH in control, and T132E knockdown TNBC cells.

**C** Immunoblot analysis of the indicated proteins in T132E-knockdown MDA-MB-231 and MDA-MB-468 cells with or without overexpression of CUL4B.

**D** Immunoblot analysis of indicated proteins in T132E-knockdown MDA-MB-231 and MDA-MB-468 cells treated with VO-OHpic or DMSO.

**E** Immunoblot analysis of indicated proteins in T132E-knockdown MDA-MB-231 and MDA-MB-468 cells treated with 740 Y-P or DMSO.

**Table S1:** The short hairpin RNA sequences used in this study.

| Gene | Target sequence |
| --- | --- |
| *TMEM132E* | TGAAGTCACTGACTAGGTC |
| *CON077* | TTCTCCGAACGTGTCACGT |

**Table S2:** The antibodies used in this study. Abbreviations: Proteintech (PTG); Cell Signaling Technology (CST); Immunoblotting (IB); Immunohistochemistry (IHC)

| Antigen | Source | Identifier | Analysis |
| --- | --- | --- | --- |
| HA | PTG | 66006-1-Ig | IB |
| HA | PTG | 51064-2-AP | IB |
| GAPDH | PTG | 60004-1-Ig | IB |
| ERα | Servicebio | GB111843 | IB |
| CUL4B | PTG | 12916-1-AP | IB |
| Phospho-Akt (Thr308) | CST | 13038 | IB |
| Phospho-Akt (Ser473) | CST | 4060 | IB |
| Akt (pan) | CST | 4685 | IB |
| PTEN | PTG | 60300-1-Ig | IB |
| Phospho-PTEN (Ser380+Thr382+Thr383） | Affinity | AF4450 | IB |
| PI3 Kinase p85alpha | ABclonal | A4992 | IB |
| Phospho-PI3-kinase p85- alpha/ gamma (Tyr467/199) | Abways | CY6428 | IB |
| CREB | CST | 9197 | IB |
| Phospho-CREB (Ser133) | CST | 9198 | IB |
| mTOR | CST | 2972 | IB |
| Phospho-mTOR (Ser2448) | CST | 2971 | IB |
| MMP2 | Santa Cruz Biotechnology | sc-13595 | IB IHC |
| MMP2 | BBI | D161446 | IB |
| MMP3 | BBI | D220095 | IB |
| SNAI1 | PTG | 13099-1-AP | IB |
| SNAI2 | Affinity Biosciences | AF4002 | IB |
| TWIST1 | PTG | 25465-1-AP | IB |
| E-cadherin | CST | 3195 | IB IHC |
| CDH1 | BBI | D260656 | IB |

**Table S3:** The primer sequences used for RT-qPCR analysis.

| Gene | Forward primer (5’–3’) | Reverse primer (5’–3’) |
| --- | --- | --- |
| *TMEM132E* | TGAAGGCCAAGAAGGGTGTG | GCTGCAAGATCTCCAAGGGG |
| *ESR1* | AGGTGCCCTACTACCTGGAG | AACGACTATATGTGTCCAGCCA |
| *CUL4B* | TATTAGTTGGCAAGAGTGCAT | CCAGTAACCCATTGTCAGGAT |
| *GAPDH* | ACAACAGCCTCAAGATCATCAG | GGTCCACCACTGACACGTTG |
